# A New Pes Planus Automatic Diagnosis Method: ViT-OELM Hybrid Modeling

**DOI:** 10.3390/diagnostics15070867

**Published:** 2025-03-28

**Authors:** Derya Avcı

**Affiliations:** Vocational School of Technical Sciences, Firat University, Elazig 23119, Turkey; davci@firat.edu.tr

**Keywords:** pes planus, vision transformer (ViT), automatic diagnosis, optimum extreme learning machine (OELM), ViT-OELM modeling

## Abstract

**Background/Objectives:** Pes planus (flat feet) is a condition characterized by flatter than normal soles of the foot. In this study, a Vision Transformer (ViT)-based deep learning architecture is proposed to automate the diagnosis of pes planus. The model analyzes foot images and classifies them into two classes, as “pes planus” and “not pes planus”. In the literature, models based on Convolutional neural networks (CNNs) can automatically perform such classification, regression, and prediction processes, but these models cannot capture long-term addictions and general conditions. **Methods:** In this study, the pes planus dataset, which is openly available on the Kaggle database, was used. This paper suggests a ViT-OELM hybrid model for automatic diagnosis from the obtained pes planus images. The suggested ViT-OELM hybrid model includes an attention mechanism for feature extraction from the pes planus images. A total of 1000 features obtained for each sample image from this attention mechanism are used as inputs for an Optimum Extreme Learning Machine (OELM) classifier using various activation functions, and are classified. **Results:** In this study, the performance of this suggested ViT-OELM hybrid model is compared with some other studies, which used the same pes planus database. These comparison results are given. The suggested ViT-OELM hybrid model was trained for binary classification. The performance metrics were computed in testing phase. The model showed 98.04% accuracy, 98.04% recall, 98.05% precision, and an F-1 score of 98.03%. **Conclusions**: Our suggested ViT-OELM hybrid model demonstrates superior performance compared to those of other studies, which used the same dataset, in the literature.

## 1. Introduction

Pes Planus is a disease commonly known as flat feet. Deep learning (DL) is increasingly used in the medical field, and has made significant contributions in the diagnosis of orthopedic disorders. These technologies are also used in the diagnosis of pes planus (flat feet). In a study conducted by Gül et al. [1], an algorithm was developed that automatically diagnoses pes planus using lateral foot radiographs of 439 patients between the ages of 14–47. In this study, features were extracted from the images using an explainable Vision Transformer (ViT), and an Optimum Extreme Learning Machine (OELM) classifier was trained with these features to classify pes planus with an accuracy rate of 98.68%. In this, deep learning structures can be used with high accuracy rates in the diagnosis of pes planus. In this way, faster and more objective diagnoses can be made in clinical settings. The importance of Artificial intelligence (AI) technology is increasing in modern medicine. AI has some advantages in the medical field. The most significant advantage is that it can more quickly and accurately make decisions using big datasets [2,3,4,5,6,7]. Medical image processing is useful in early disease diagnosis and treatment. For this purpose, Computer-Aided Diagnosis (CAD) systems are used, especially in the area of medical image processing. Computer Aided Diagnosis systems are of vital significance in medical imaging-based early diseases diagnostics [8]. The aim of these CAD systems is automatic medical image processing. These automatic medical image processors can be used to analyze medical X-rays, MRIs, and CT images. These automatic medical image processing systems use trained algorithms that employ deep learning structures for analyzing medical images. By using these analysis results, abnormalities and disease symptoms can be diagnosed [9,10]. Furthermore, disease diagnostic systems based on Artificial intelligence (AI) can assist in reducing global health disparities by improving access to healthcare services. AI systems are used some areas in medicine, such as are image analysis and early diagnosis [11].

Gul et al. [1] proposes an innovative structure based on CNN-SVM for early diagnosis of pes planus disease using an X-ray pes planus images dataset. Within the scope of the study, a new weight-bearing X-ray dataset labeled by radiologists was collected. In this study, X-ray pes planus images were augmented to achieve a higher accuracy ratio. Then, these images were divided into 4 and 16 parts with dimensions of 256 × 256 × 3 and 128 × 128 × 3 patches, respectively. Finally, at the end of this process, a total of 21 pes planus images were obtained for each X-ray pes planus image (20 parts + 1 original image). The proposed model achieved an accuracy rate of 95.14%, demonstrating that it can be utilized as an assistant tool for early diagnosis of pes planus disease in clinical applications.

Danaci et al. [2] provides a strong example for the use of AI in medical diagnosis, particularly in the detection of pes planus (flat feet) from X-ray pes planus images. The study’s methodology appears robust; feature extraction, selection, and optimization techniques were used to improve classification accuracy. Using multiple feature extraction methods (Relief-F, Lasso, and RFE) and classifiers (KNN, SVM, DT, and XGBoost) increases the reliability of the study, with the highest success rate being achieved by the XGBoost algorithm with 97% accuracy.

Doğan et al. [12] used Vgg16, ViT-B/16, and Vgg16-ViT models separately for pes planus classification. In here, it was computed 96.8% accuracy.

This study aimed for the automatic diagnosis of pes planus disease using X-ray pes planus images. A new OELM-ViT hybrid model based on AI structures is suggested. It is possible to use a Vision Transformer (ViT) for the early diagnosis of pes planus (flat feet) disease, utilizing deep learning techniques to automate the diagnosis by analyzing imaging, such as X-rays of the sole of the foot or pedobarography. The structure of Vision Transformer (ViT) divides the features into small distributions (patches) and separates them into a series, and then analyzes them with attention division. ViT contains 1000 features for each copy plan image. Then, pes planus images and not pes planus images are classified using some AI structures. The target of this work is to form a more effective architecture for the early diagnosis of pes planus disease.

In Section 2, the material and methods, the image classification method based on the Vision Transformer (ViT), and the Optimum Extreme Learning Machine classifier are described. In Section 3, the applications, application setup, and performance metrics are presented. In Section 4, the Discussion and conclusions are presented. In this section, the training and test pes planus image datasets are evaluated according to the performance and evaluation criteria of the proposed OELM-ViT model.

Novelties of This Study:

- X-ray Images and Dataset: It is stated in the literature that there are only two studies that share a dataset composed of X-ray pes planus images for automatic flatfoot diagnosis. This indicates the importance of composing X-ray pes planus image datasets for flatfoot diagnosis in this field.
- ViT-OELM Model: In this study, the Optimum Extreme Learning Machine (OELM) head layer was added to the basic ViT architecture model instead of the Multi-Layer Perceptron (MLP)’s head layer being used. The suggested ViT-OELM model comprises 12 encoder blocks. This developed ViT-OELM architecture, with an input size of (224, 224), allows for input images of 224 × 224 pixels. The proposed ViT-OELM model reduces computational load and increases accuracy by using a more comprehensive feature selection method. As a result, a more successful model for flatfoot diagnosis has been developed. The performance results of this proposed ViT-OELM model are superior to other deep learning structures.
- Feature Extraction and Selection: In feature extraction, experiments conducted on raw data show that accuracy increases, and high accuracy rates are achieved. This demonstrates that effective feature extraction increases the high accuracy ratio and contributes to quickly and correctly creating the architecture.
- Classification: By using OELM as classifier in this proposed ViT-OELM hybrid modeling, some classification advantages are obtained. These are the following:
  - Faster Training: OELM is faster than fully connected layers and traditional neural networks because it optimizes only the output weights.
  - Higher Accuracy: Provides better classification success with optimum weight selection and kernel functions.
  - Reduces Over-Learning: The generalization ability of the model is increased with regularization methods.
- Optimization: By using the Adam algorithm as an optimizer in this proposed ViT-OELM hybrid model, some optimization advantages are obtained. These are the following:
  - Parameter updates are more balanced thanks to momentum and adaptive learning rate.
  - Weights and bias values are optimized to prevent over-learning.
  - Faster convergence rates are provided, which is especially useful when working with large datasets.
- Main Contributions: The new ViT-OELM hybrid model using X-ray pes planus images presented in this study can be a flexible instrument for the early diagnosis of flatfoot and can obtain high accuracy success. The ViT-OELM hybrid model can be used in clinical applications thanks to its high classification accuracy.

## 2. Materials and Methods

**Figure 1:**
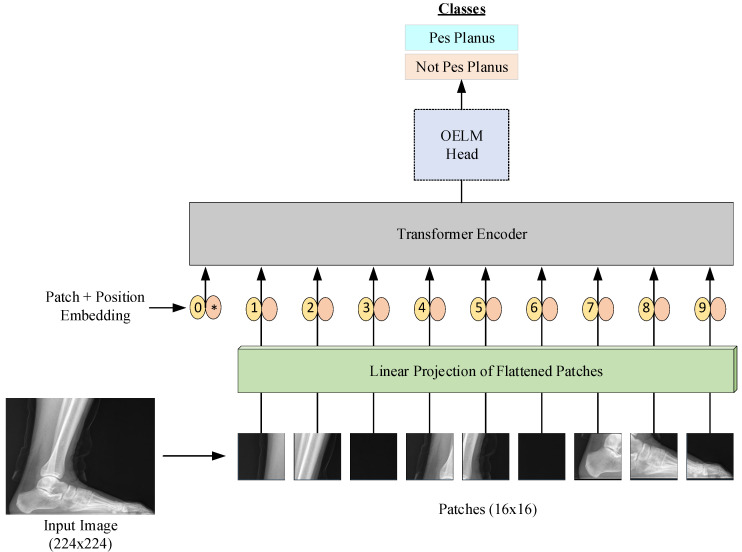
Block diagram of our suggested approach using the ViT-OELM for pes planus diagnosis. In here, numbers and * represent patch and position respectively.

This work suggests an advanced deep learning approach for pes planus diagnosis using a Vision Transformer–Optimum Extreme Learning Machine (ViT-OELM) hybrid model. The ViT-OELM hybrid model is trained on a pes planus image dataset, enabling it to accurately classify images into various subtypes. Given a pes planus image, the model predicts the image subtype using deep learning techniques. The suggested ViT-OELM hybrid model is shown in Figure 1.

The steps in Figure 1 can be explained as follows:

This diagram shows a Vision Transformer (ViT) model that classifies X-ray foot images as “Pes Planus” (flat feet) and “Not Pes Planus” (not flat feet). The detailed description of the workflow is as follows:

- Input Image (224 × 224 resolution): The process starts with a foot X-ray.
- Patch Extraction (16 × 16 patches): The image is divided into smaller patches of 16 × 16 pixels.
- Linear Projection of Flattened Patches: Each patch is flattened into a vector and projected into a higher dimensional space.
- Patch + Position Embedding Process: Position embeddings are added to the patch embeddings to preserve the positional information.
- Transformer Encoder: The embedded patch array is processed by a Transformer encoder.
- OELM Header: The encoded feature representation is transferred to the OELM (Optimized Extreme Learning Machine) header and the classification process is performed.
- Classification Output: The final classification output determines whether the X-ray image is “Pes Planus” (flat foot) or “Not Pes Planus” (not flat foot).

### 2.1. Used Pes Planus Image Dataset

The pes planus dataset was composed by Gül et al. [1]. Then, this pes planus dataset was submitted for open access on the Kaggle database for storage [3]. The pes planus image dataset contains 842 numbered X-ray pes planus images from 439 numbered persons. These patients, who applied to the Radiology Clinic of Elazığ Fethi Sekin City Hospital with suspected pes planus, were from 14 to 47 years old. These pes planus image dataset were obtained using a Philips dual-detector digital X-ray device (65 kV, 6.3 mAs) and stored in JPG format in the data repository.

When creating the pes planus image dataset, persons with known neurological diseases, individuals with acute orthopedic trauma, or those who had previously undergone lower extremity orthopedic surgery were excluded. X-ray pes planus images of 18 persons with flat feet in the collected dataset were removed because of issues with low quality and low resolutions.

**Figure 2:**
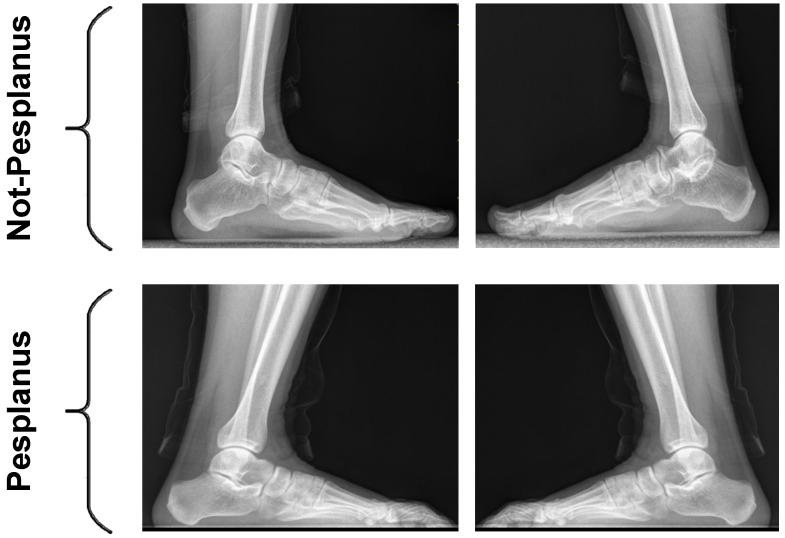
Example images from both pes planus and not pes planus classes in the dataset.

To compute the calcaneal tilt angle, the remaining 842 X-ray pes planus images of 421 persons were labeled by radiologists. If individuals had a calcaneal angle under 18 degrees, they were determined to have “pes planus” (flat feet). If an individual had an angle above 18 degrees, they were determined to be “not pes planus”. After labeling, 402 of the 842 X-ray pes planus images were catagorized as pes planus. A total of 440 of the 842 X-ray pes planus images were catagorized as not pes planus [1,2,3]. Figure 2 shows example images from both classes, which are “pes planus” and “not pes planus”, in the pes planus image dataset.

### 2.2. Image Classification Method Based on Vision Transformers (ViTs)

**Figure 3:**
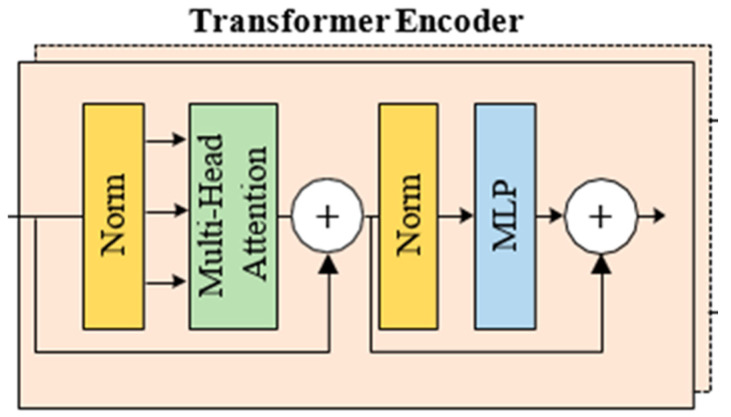
Block diagram of the Transformer encoder–decoder.

Vision Transformers (ViTs) are an advanced deep learning stucture. They are commonly used in natural language processing (NLP) for composing large language models [13]. There is an attention mechanism in the ViT architecture. This mechanism is also used in the suggested new ViT-OELM hybrid model to evaluate the significance of various parts of the input when carrying out predictions. The attention mechanism obtains the weighted sum of the input values according to a given location or query [13,14,15]. In ViTs and NLP, the distribution of self-attention rates is estimated by the distributions of each input with the other members. NLP uses relationships between words. ViTs use relationsships between pixels or image patches. In addition, the Transformer uses an encoder–decoder structure [16,17,18]. This structure takes the input, and produces a set of hidden descriptions [19,20,21]. The block diagram of the Transformer encoder–decoder structure is given in Figure 3.

**Figure 4:**
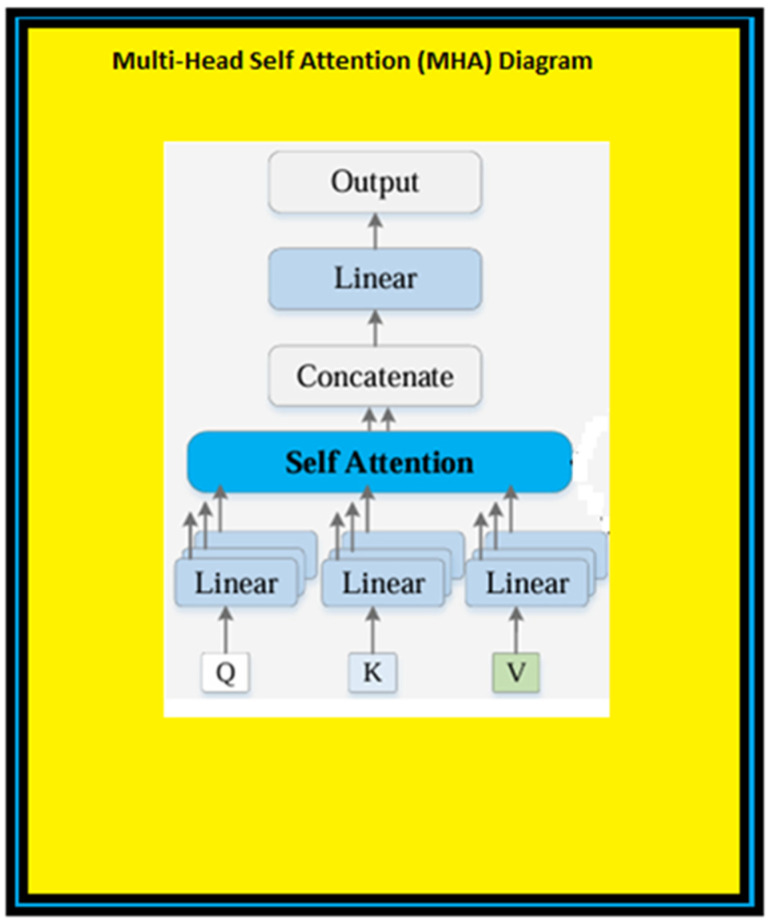
Block diagram of the MHA mechanism.

**Figure 5:**
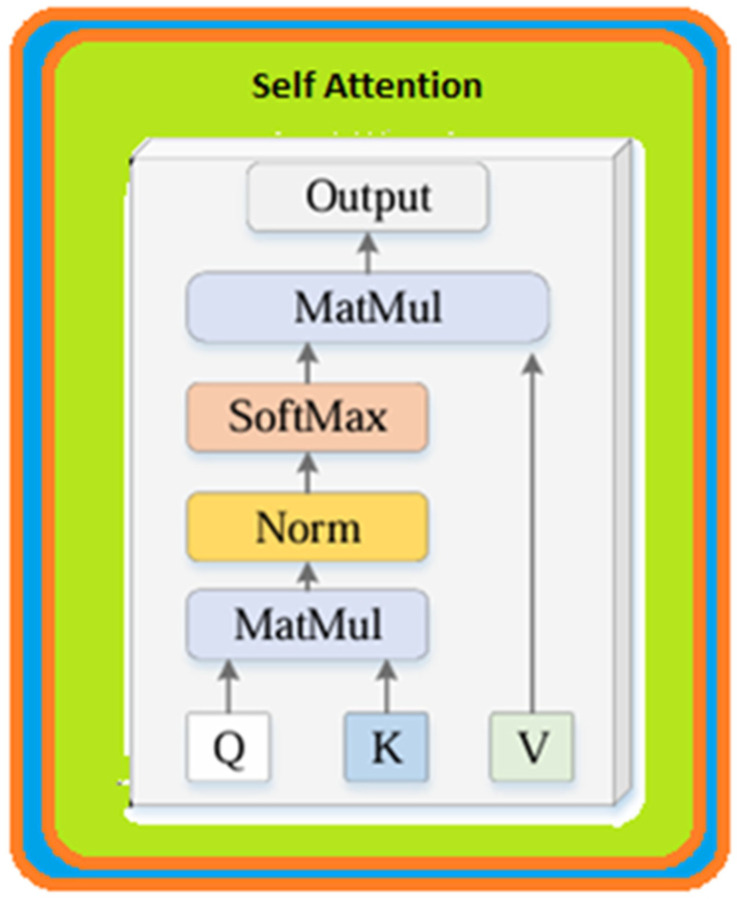
Block diagram of the attention mechanism used in this paper.

This set of representations is sent to the decoder to compose the output. Both the encoder and the decoder consist of multilayered structures containing multiple attention mechanisms. This structure helps the suggested new ViT-OELM hybrid model to learn and extract information from the input at various levels of summary; thereby, the sense of the input can be understood. Figure 4 describes the use of multi-headed attention (MHA). A block diagram showing the attention mechanism used in this paper is shown in Figure 5.

In MHA, each patch of the input vector is subjected to an attention operation and transformed into three different vectors using the following weight matrices: query (Q), key (K), and value (V) [22]. The importance of each patch is determined by computing the dot product of the query and key vectors, and a score matrix. Then, attention weights are generated by applying the softmax activation function to the score matrix. Then, to obtain the attention output, the attention weights are multiplied by the value vector.

Then, the attention mechanism for each patch and the resulting attention matrices are calculated. The attention mechanisms are summed and realized by a linear layer. Later, a regression head is used to produce the output of the MHA mechanism. The Vision Transformer model, consisting of attention blocks and MLP (Figure 5), was proposed by Dosovitskiy et al. [23]. ViTs process images as input differently than standard Transformers. Principally, Vision Transformers separate images into smaller patches. Then, they use a Transformer structure for each of these patches [24]. Based on this, the suggested new ViT-OELM hybrid model learned and extracted information by separating images into multiple levels. In NLP, Transformer architecture works similarly to this. One of the important features of ViTs is that they do not need any preprocessing steps such as, for example, convolutional layers. Instead of preprocessing steps, ViTs separate the images into tiny patches. Then, they use a Transformer structure for each of these patches [25]. The linear projection in ViTs reduces the size of the image patches before entering them into the Transformer network. Although this situation may seem negative, this linear projection plays an important role in the suggested ViT-OELM architecture. This status provides the suggested ViT-OELM architecture with a more effective model, because the suggested ViT-OELM architecture can be trained on larger images by decreasing the computational load due to this advantage of linear projection.

In this study, the Optimum Extreme Learning Machine (OELM) head layer was added to a basic ViT architecture model instead of a Multi-Layer Perceptron (MLP) head layer being used. The suggested ViT-OELM model is composed of 12 number-encoder blocks. Readers may find more detailed information in [26].

The 1000 features that come out of the VİT (Vision Transformer) model are usually a representation of the features learned during the training phase of the model. Vision Transformers use a different approach to image processing compared to traditional convolutional neural networks (CNNs). In the VİT-OELM hybrid model used in this study, feature selection and extraction are performed in the following stages:

1. Creating Patching: The image was first divided into small pieces (patches). Each patch has a fixed size of 16 × 16 pixels. These patches were used as the input for the VİT-OELM hybrid model.
2. Linear Patch Embeddings: Each patch was transformed into a vector. Here, a linear projection (linear transformation) was used to create a feature vector representing each pixel’s information in the patch.
3. Transformer Encoder: These features were then passed through Transformer encoder layers. Here, by using the attention (self-attention) mechanism, the model learns the relationship of each patch to the other patches and creates more meaningful feature representations.
4. Inference and Classification: Through the Transformer layers, the features of each patch are combined, and the VİT-OELM hybrid model creates a higher-level representation. As the output of the VİT-OELM hybrid model, 1000 features obtained for each image data are set to input for the OELM head classifier.

This developed ViT-OELM hybrid architecture, with an input size of (224, 224), confirms an input image of 224 × 224 pixels. It then progresses with a Conv2D (Projection) layer, which divides the input image into small patches. These patches are portions of the image. The Conv2D layer extracts the patches using a 16 × 16 filter. The ViT-OELM performs a transformation on each patch using the Conv2D layer. The sizes of the blocks entering and exiting each encoder block are the same (1, 197, 768). Encoder blocks include an attention ratio, an intermediate layer, and an output range. Attention ranges are used to extract features in the ViT model and to specify important features. These intermediate layers process richer features from the image to produce higher-level representations. This intermediate layer includes a Dense layer and an activation function named GELU Activation. The output layer is resized to the original dimensions at the output of the intermediate layer, and contains a Dropout layer. One of the LayerNorm layers is utilized before the input. The other LayerNorm layer operates after the output of each encoder variation. LayerNorm realizes the normalization process for the output. Here, LayerNorm implements the weighted normalization operations. This ability of ViTs makes the model’s work more stable and robust. The normalization system found after Encoder Block-12 normalizes the data in (1, 768) dimensions and gives an output of (1, 2) results. After, by using a Linear layer, these data are converted into (1, 2) piecewise class labels, which are pes planus and not pes planus. Thus, predictions can be made for 2 various classes of pes planus diagnosis.

### 2.3. Optimum Extreme Learning Machine Classifier

OELM (Optimized Extreme Learning Machine) is an algorithm used in machine learning and especially in classification problems. Some advantages of OELM over SVM (Support Vector Machine), XGBoost, and some other classification algorithms are as follows:

1. Speed and Computational Efficiency: OELM is a model that does not require parameter adjustments during its training. This provides faster training times compared to algorithms such as SVM or XGBoost. OELM uses a single-layer artificial neural network, and training this structure is usually faster. Algorithms such as SVM or XGBoost can be more complex in terms of optimizing parameters and the training time of the model.
2. Less Parameter Tuning Required: OELM offers a fast training process by assigning random weights only for the input layer. The model generally does not require hyperparameter adjustments, which increases its ease of use. Algorithms such as SVM and XGBoost require more parameter tuning. For example, parameters such as kernel type, C and gamma parameters for SVM, and learning rate and number of trees for XGBoost need to be optimized.
3. General Performance: OELM generally has good results on small and medium-sized datasets. Especially on large datasets, methods such as SVM or XGBoost can be more powerful. SVM and XGBoost can generally give better results than OELM in nonlinear classifications, but OELM has great advantages in terms of fast learning and implementation.
4. Risk of Overfitting: OELM provides fast learning to reduce the risk of overfitting, and can increase generalization ability by assigning random weights during the training process. SVM and XGBoost may have risks of overfitting if they are not tuned correctly, since they have more parameters.
5. Complexity and Flexibility: Since the architecture of OELM is simple, good results can be obtained with less complexity in some cases. However, in more complex and high-accuracy tasks, algorithms such as SVM or XGBoost can be more flexible and powerful.

In summary, the advantages of OELM are generally related to fast training times, low parameter tuning requirements, and low computational costs. However, in very large and complex datasets, algorithms such as SVM or XGBoost can perform more powerfully.

For these reasons, OELM was preferred over SVM, XGBoost, or other classifiers in this study.

Unlike traditional neural networks, ELM is a method that can train quickly by optimizing only the output weights [27,28]. Its main features are:

- Input weights are randomly assigned and not changed.
- Only the weights in the output layer are optimized.
- It provides fast learning and works efficiently on large datasets.

However, ELM has some disadvantages:

- Randomly determined weights can decrease the accuracy performance of the model.
- It may carry the risk of overfitting.
- It may require sensitive hyperparameter adjustments.

The Optimum Extreme Learning Machine (OELM) is an optimized version of the ELM algorithm. ELM is a training algorithm developed for feedforward neural networks (FNN) with a single hidden layer, which provides a very fast training process with randomly determined weights. OELM is an optimized version of the traditional ELM model which improves on some of its weaknesses. In standard ELM, hidden layer weights and bias values are randomly determined, and only the output weights are obtained with a closed-form solution. However, this random selection can lead to problems such as instability and low generalization performance in some cases.

The basic features of the OELM are given below:

- Optimization of Weights

OELM aims to increase the accuracy and generalization ability of the suggested ViT-OELM hybrid model by determining more optimized hidden layer weights. Instead of randomly selected input weights in standard ELM, better weight values are determined using optimization algorithms.

- Better Generalization Ability

Optimum weight selections are made to prevent overfitting to the dataset. The accuracy rate of the suggested ViT-OELM model increases, and it makes more successful predictions in unknown data.

- Fast Learning

One of the biggest advantages of ELM, its fast learning ability, is preserved. Faster results are obtained with fewer errors due to optimal weight selection.

- More Stable Results

Since optimized weights are used instead of random weights, more stable results are obtained, rather than variable results in different trainings.

OELM is a version developed to overcome the disadvantages of ELM. The word optimum means that the model is optimized to give the best performance. The following techniques are generally used in OELM:

1. Optimal Weight Initialization: A more optimized initial weight matrix is used instead of a randomized one. The best weights can be selected with methods such as the Adam Algorithm (AA) [29], Genetic Algorithms (GAs) [30], Particle Swarm Optimization (PSO) [31], or Grid Search [32].
2. Stronger Regularization: Over-learning is prevented by adding L1 or L2 regularization.
3. Kernel-Based OELM (K-OELM): It provides better generalization in nonlinear problems by using kernel functions.
4. Hyperparameter Optimization: Methods such as Grid Search, Bayesian Optimization or Evolutionary Algorithms can be used to determine the best hyperparameters of the model.

The advantages of the OELM are given below:

- Higher accuracy: It can generalize better compared to ELM.
- Fast training process: It is faster than traditional neural networks.
- Reduces over-learning: The model becomes more stable with regularization and optimization methods.

The VIT-OELM (Vision Transformer-based Optimum Extreme Learning Machine) model is an artificial intelligence model that combines the components of Vision Transformer (ViT) and Optimum Extreme Learning Machine (OELM). The hyperparameter selection and optimization process of this model generally includes the following steps:

Flow Steps:

1. Start: Input data are prepared. Hyperparameters (lambda, number of hidden layers) are determined.
2. Data Preprocessing: Input data are normalized. They are divided into training and test datasets.
3. Random Assignment of Hidden Layer Weights: The weights and bias values of the hidden layer are randomly assigned.
4. Calculation of Hidden Layer Output: The activation function is applied.
5. Regularization and Hyperparameter Optimization: The regularization parameter λ (lambda) is selected. The number of hidden layers is optimized.
6. Calculating Output Weights (Adam): Weight updates are performed with Adam optimization instead of the Moore–Penrose Pseudo-Inverse.
7. Weight Update with Adam Optimization: The learning rate, beta values, etc., are determined. The first moment (m) and second moment (v) are calculated. Weights are updated.
8. Calculating Loss Function: MSE or another error metric is calculated.
9. Checking Stopping Criteria: Has the maximum iteration or specified error threshold been reached?
  Yes: Proceed to testing phase. No: Return to Adam optimization.
10. Testing the Model: Model performance is measured with test data.
11. Finish: The trained model is saved and the results are reported. With this method, the ViT-OELM hybrid model provided high accuracy and fast calculation advantages in pes planus image classification.

**Figure 6:**
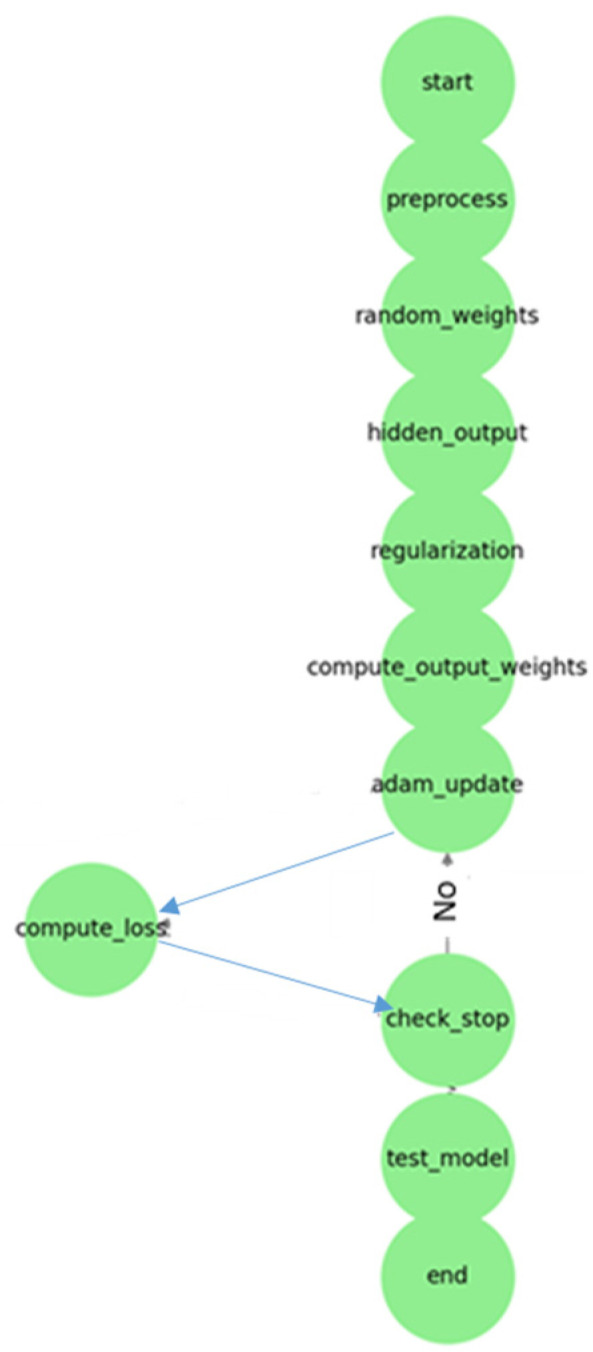
The hyperparameter selection and optimization process of the suggested ViT-OELM hybrid model.

As below, the hyperparameter selection and optimization process of the suggested ViT-OELM hybrid model is shown in Figure 6.

OELMs can be an effective method in image classification, biomedical data analysis, time series prediction, and many other machine learning problems. Here, the OELM is used to improve the classification performance of the ViT model. OELMs can perform faster and more accurate classifications by better generalizing the features extracted from the ViT model. ViTs process each patch as a series of vectors by dividing the images into small pieces (patches). Meaningful features are extracted from these vectors through encoder blocks. Normally, a fully connected classifier is located in the last layer of ViT. Instead, the accuracy of the model can be increased by adding an OELM-based classifier.

## 3. Results

This study presents an advanced ViT-OELM hybrid model for increasing the accurate identification and classification of pes planus images. This approach uses a pes planus images dataset [3] to train the suggested ViT-OELM hybrid model, thus enabling the model to classify these images with high accuracy. The proposed ViT-OELM hybrid model takes a pes planus image as input and infers pes planus diagnosis using its deep learning capabilities. The suggested ViT-OELM hybrid model is explained in Figure 1.

Here, a new ViT-OELM hybrid Modeling is proposed for pes planus diagnosis due to the advantages, mentioned above, of both ViT and OELM. The developed ViT-OELM hybrid approach used is described in Figure 1.

1. Definition of Dataset

**Figure 7:**
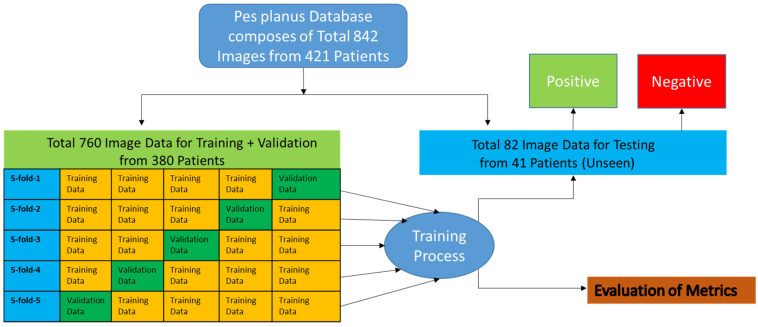
The training process for the suggested VİT-OELM hybrid model.

As indicated in Figure 1, a total of 842 pes planus (flat feet) X-ray images taken from 421 patients were used. The images were arranged in an average of 2 per patient. According to the opinions and recommendations of the referees of this image database, 380 images out of a total of 760 were used for training, and to progress healthily, 82 images taken from 41 patients which were classified as pes planus or not pes planus were also marked for testing. In Figure 7, the training process for the suggested VİT-OELM hybrid model is given.

2. Dataset Division (K-Fold Cross Validation)

To evaluate the performance of the model, as mentioned above, a total of 760 data from 380 patients were separated into training and validation sets, and were used with the 5-fold cross-validation method. From there, 82 image data from 41 patients separated for testing were excluded from this 5-fold cross-validation to prevent any data leakage.

- The dataset was divided into five equal parts.
- In each iteration, one part of the data was used for validation, and the remaining four parts were used for training. Thus, a total of 1520 data were used for training and 380 data were used for validation over five iterations.
- The model was trained from scratch in each layer (fold), and the average performance metrics were reported.

Thanks to this method, how the model performed on different data groups was measured more reliably.

### Application Setup and Performance Metrics

In our suggested ViT-OELM hybrid model, the pre-learned model ‘vit-base-patch16-224’ was used, due to the high cost of training a ViT model with random weights. This model was pre-trained with the ImageNet-21k (14 million images, 21,843 classes) dataset. Here, the Timm and PyTorch 2.2.0 libraries were used in the application of the model. The output distribution of the model was changed according to the changes in the classes, which are pes planus and not pes planus, in the dataset.

Firstly, the input pes planus images were resized to fit the ViT-OELM model in the preprocessing stage. Since the default input size of the ViT-OELM model is 224 × 224, all the examples in the dataset are converted to this size.

The ViT-OELM hybrid model classified the pes planus data, which included 402 pieces as “pes planus” and 440 pieces as “not pes planus” including a total of 842 data [1,2,3]. For this purpose, the OELM layer was added to the basic ViT architecture model instead of using the MLP layer [26]. The ViT-OELM hybrid model consists of 12 encoder blocks. This developed ViT-OELM architecture, with an input size of (224, 224), confirms an input pes planus image of 224 × 224 pixels. It then begins with a Conv2D (Projection) layer, which divides the input image into a series of small patches. These patches are typically called “patches”, and each represents a portion of the image. The Conv2D layer extracts the patches using a 16 × 16 filter. The ViT-OELM hybrid model performs a transformation on each patch using the Conv2D layer. These transformations convert the image portions into a series of vector representations, which are processed in the encoder blocks of the ViT-OELM hybrid model. As explained in detail in Section 2.2, features taken from the ViT output are given to the inputs of the OELM classifiers. For Optimization ELM classifier parameters (hidden layer weights (W), hidden layer bias (b), output weights (β\betaβ), and regularization parameters (λ\lambdaλ)) to update to a better fit, the Adams programming and the CrossEntropyLoss loss function were used during the training of the OELM. By using the Adam algorithm as an optimizer in this proposed ViT-OELM model, some optimization advantages are obtained. These are the following:

- Parameter updates are more balanced, thanks to momentum and the adaptive learning rate.
- Weights and bias values are optimized to prevent over-learning.
- Provides faster convergence, especially useful when working with large datasets.

The batch size value of the ViT-OELM model is 16 of the OELM’s. The learning rate of the ViT-OELM model is 0.0001 during the training of the OELM. The ViT-OELM model requires 250 training epochs, but the early stop (early stop) function can be used to monitor losses. If the error does not decrease over 10 sequential epochs, the suggested ViT-OELM model’s training will be terminated, and the weights of the Epoch that provided the highest efficiency will be saved.

This effect was determined by a trial and error (brute force) approach, and the values that gave the best results on the continuous dataset were selected. All experiments with the determined effect were carried out in the Google Colab environment.

**Figure 8:**
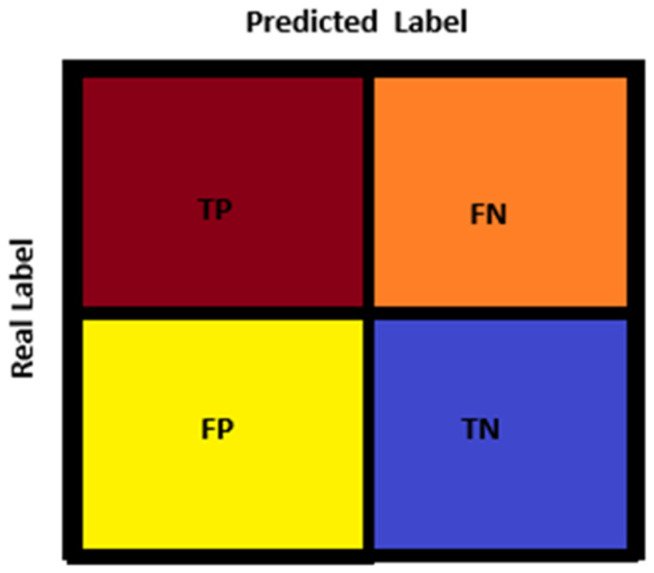
Confusion matrix for binary-class scenario.

For the evaluation of the results of the proposed ViT-OELM model in its tasks, metrics based on the complexity matrix were used in this study. A complexity matrix shows the distribution between the class label predicted by the model for a given input pes planus image and the true class label of this pes planus image. In AI studies, different situations can emerge in large layers of the used models. Four metrics are used: True Positive (TP), True Negative (TN), False Positive (FP) and False Negative (FN). Figure 8 shows how these metrics are placed in a random class option for binary classifications. The complexity matrix for binary classification (binary classification) can be shown as follows:

Here, the evaluation of the proposed ViT-OELM hybrid model was carried out using different performance metrics, which are accuracy, precision, recall, and F1 score, respectively. These metrics validate information about the ViT-OELM hybrid model’s performance from different perspectives.

- Calculated metric values of the proposed ViT-OELM model:

(1) $$\mathrm{Accuracy}\;(\mathrm{Acc})=\frac{\left(TP+TN\right)}{\left(TP+FP+FN+TN\right)},$$

(2) $$\mathrm{Sensitivity}=\;\frac{TP}{TP+FN},$$

(3) $$\mathrm{Specificity}=\;\frac{TN}{TN+FP},$$

(4) $$\mathrm{Recall}\;(\mathrm{Rec})=\frac{TP}{TP+FN},$$

(5) $$\mathrm{Precision}\;(\mathrm{Pre})=\frac{TP}{TP+FP},$$

(6) $$F-1\;\mathrm{Score}\;(F1)=\frac{\left(2\times \left(Pre\times Rec\right)\right)}{\left(Pre+Rec\right)}$$

In the results of the ViT-OELM model’s testing phase, the most suitable values of TP, TN, FP, and FN were found as 201, 201, 4, and 4, respectively. The Metric value of the proposed ViT-OELM model is presented in Table 1. The overall performance of the model was determined by calculating the average accuracy for five folds. In this study, the deep learning model developed for pes planus diagnosis was tested with 5-fold cross-validation and a reliable result was obtained. Finally, the performance of the model was analyzed in detail using different metrics.

Since a pre-trained AI architecture performs from an already optimized starting point, training time is reduced, and computational resources are saved compared to training architectures from scratch. Such architectures generally outperform models trained from scratch, because they have already learned general representations on a large dataset, providing a strong basis for adaptation to the target task.

**Figure 9:**
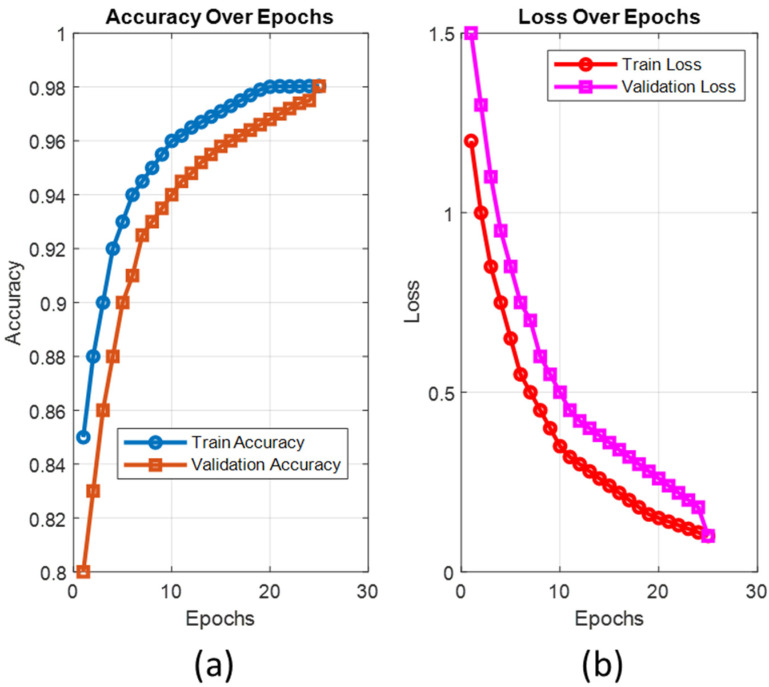
The ViT-OELM hybrid model’s performance curves. (**a**) Accuracy values. (**b**) Loss values.

Using pre-trained AI architectures, the training time for the ViT-OELM hybrid model was set to a maximum of 200 epochs. Nevertheless, due to the activation of the early stopping function, the training ended at epoch 25, and the weights were saved in ‘.pth’ format. In the training process, each epoch resulted in an average time of 102 s. The accuracy values and loss values obtained during the training phase of the suggested ViT-OELM model are given in Figure 9.

Pes planus datasets were used with the same data splitting ratios and hyperparameters used in the two-class classification task. The suggested ViT-OELM model achieved 98.04% accuracy on the test samples, demonstrating an ability to distinguish between “pes planus” or “not pes planus”. Figure 8 shows the confusion matrix and ROC curve of the test results. The ViT-OELM hybrid model, in particular, obtained 98.04% validation accuracy and 0.07% validation loss at the end of epoch 25. At the same time, tests of the pes planus images database were evaluated to obtain the proposed ViT-OELM hybrid model’s ability to generalize to new and unseen scenarios. This is an important factor for practical applications. In Figure 9, the ViT-OELM hybrid model’s performance curves can be seen. In (a), accuracy values can be seen. In (b), loss values are shown.

In Table 2, the metrics of each of the five folds, separate from the ViT-OELM hybrid model’s performance in “pes planus” or “not pes planus” classifications, are given.

In Table 3, the metrics for the total 5-fold testing data of the ViT-OELM hybrid model’s performance in “pes planus” or “not pes planus” classifications are given.

**Figure 10:**
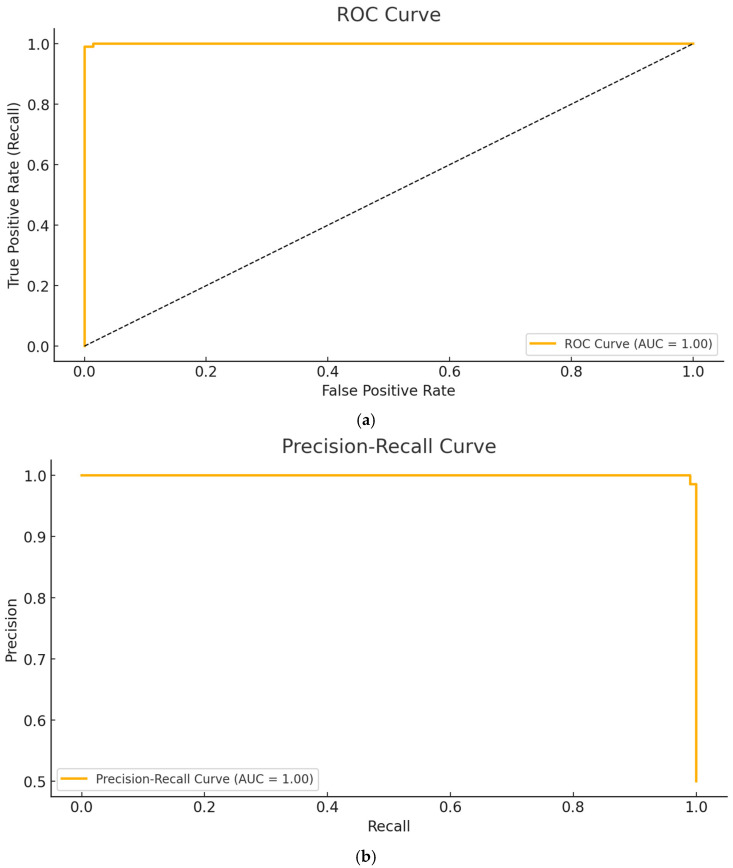
(**a**) ROC Curve, (**b**) Precision–Recall Curve.

In Figure 10, the (a) ROC Curve and the (b) Precision–Recall Curve of the ViT-OELM hybrid model testing performances were shown.

## 4. Discussion

In this paper, classification of images as pes planus and not pes planus images was carried out for the early diagnosis of pes planus disease, using a suggested new ViT-OELM hybrid model. Although pes planus diagnosis is a process that can be realized by radiologists in laboratory conditions, automating basic problems with machine learning is valuable in many areas, including in health fields. In this paper, a new ViT-OELM hybrid model, which has become popular recently, is suggested, and examined in the context of pes planus diagnosis. Table 4 presents the results of some deep learning studies using the same pes planus database in a comparative manner.

Gul et al. [1] proposed an innovative CNN-SVM based model for automatic pes planus diagnosis using X-ray pes planus images. Within the scope of the study, a new weight-bearing X-ray pes planus image dataset, labeled by expert radiologists, was collected. X-ray pes planus images were augmented. Then, they were divided into 4 and 16 parts with dimensions of 256 × 256 × 3 and 128 × 128 × 3. Here, a total of 21 images were composed for each X-ray pes planus image (20 parts + 1 original image). The suggested model obtained an accuracy rate of 95.14%, demonstrating that it can be used as an assistance tool for early diagnosing of pes planus disease in clinical applications.

Danaci et al. [2] provides a strong example of the use of AI in medical diagnosis, particularly in the detection of pes planus (flat feet) from X-ray pes planus images. Their study’s methodology appears robust; feature extraction, selection, and optimization techniques were used to improve classification accuracy. Using multiple feature extraction methods and classifiers increases the reliability of the study, with the highest success rate being achieved by the CNN-XGBoost algorithm, with 97% accuracy.

Doğan et al. [12] compared the performance of Vgg16, ViT-B/16 Vgg16-ViT models separately for pes planus detection. The highest success rate was obtained by combining the Vgg16 and ViT-B/16 algorithms, with 96.8% accuracy.

In this paper, a new ViT-OELM model was used for automatic diagnosis of pes planus. The proposed ViT-OELM model trained for two various types of pes planus database images, and obtained 98.04% accuracy. Compared to the studies shown in Table 4, higher accuracy was achieved. The superior performance of the ViT-OELM hybrid model can be associated with its unique architecture, which captures long-term dependencies between various parts of the pes planus images. This status results in better pes planus image classification performance.

The novelties of the proposed ViT-OELM hybrid model can be described as follows:

- The suggested ViT-OELM hybrid model is based on the ViT architecture, which is a popular area in AI. To demonstrate the superiority of the proposed ViT-OELM hybrid model over other deep learning models, which used the same pes planus image database, an application was created here for pes planus classification.
- The ViT-OELM hybrid model was trained on bigger pes planus image datasets; this model achieved successful results in pes planus diagnosis problems with low training costs.
- In this ViT-OELM hybrid model, the ViT and OELM hybrid architecture is different from an end-to-end Transformer structure. In this study, the Optimum Extreme Learning Machine (OELM) head layer was added to basic ViT architecture model instead of Multi-Layer Perceptron (MLP) head layer being used. As shown in Table 2, the performance results of this suggested ViT-OELM hybrid model are superior to other deep learning structures.
- The ViT-OELM hybrid model has the potential to be used in clinical applications thanks to its high classification accuracy.
- By using OELM as classifier in this proposed ViT-OELM hybrid modeling, some classification advantages are obtained. OELM is faster than fully connected layers and traditional neural networks, because it optimizes only the output weights, and it provides better classification success with optimum weight selection and kernel functions. The generalization ability of the model is increased with regularization methods.

This study provides a strong example of the use of Vision Transformer (ViT) models for medical image analysis, particularly in pes planus diagnosis. High accuracy rates demonstrate the successful performance of the suggested ViT-OELM hybrid model. In future studies, this model will be supported with the explainability element provided by Score-CAM heatmaps, increases the reliability of the model.

## Figures and Tables

**Table 1:** The metric value of the proposed ViT-OELM model.

Number of 5-Fold	Accuarcy (%)	Sensitivity (%)	Specificity (%)	Precision (%)	F-1 Score (%)
5-fold-1	96.34	95.12	97.56	97.50	96.29
5-fold-2	98.78	100	98.78	97.61	98.79
5-fold-3	98.78	100	98.78	97.61	98.79
5-fold-4	97.56	97.56	97.56	97.56	97.55
5-fold-5	98.78	97.56	100	100	98.76
Mean ± Std	98.04 ± 0.976	98.04 ± 1.826	98.53 ± 0.913	98.05 ± 0.973	98.03 ± 0.995

**Table 2:** The metrics of each of the five folds, separate from the ViT-OELM hybrid model’s performance.

5-Fold-1		5-Fold-2		5-Fold-3		5-Fold-4		5-Fold-5	
TP: 39	FN: 2	TP: 41	FN: 0	TP: 41	FN: 0	TP: 40	FN: 1	TP: 40	FN: 1
FP: 1	TN: 40	FP: 1	TN: 40	FP: 1	TN: 40	FP: 1	TN: 40	FP: 0	TN: 41

**Table 3:** The metrics of the total 5-fold testing data of the ViT-OELM hybrid model’s performance.

Metrics of Total 5-Fold Testing Data	
TP: 201	FN: 4
FP: 4	TN: 201

**Table 4:** Comparison of results of studies using the same pes planus database.

St	Year	Metod	Accuracy (%)
Gul et al. [1]	2023	CNN-SVM	95.14
Doğan et al. [12]	2024	Vgg16-ViT-B/16	96.8
Danaci et al. [2]	2025	CNN-XGBoost	97
This study	2025	ViT-OELM	98.04

## Data Availability

Data is available at https://www.kaggle.com/datasets/suleyman32/pesplanus-two-class-dataset (accessed on 10 January 2025).

## References

[1] Gül Y., Yaman S., Avcı D., Çilengir A.H., Balaban M., Güler H. (2023). A novel deep transfer learning-based approach for automated Pes Planus diagnosis using X-ray image. Diagnostics.

[2] Danaci C., Avci D., Tuncer S.A. (2025). Diagnosis of pes planus from X-ray images: Enhanced feature selection with deep learning and machine learning techniques. Biomed. Signal Process. Control..

[3] Gül Y., Yaman S., Avcı D., Çilengir A.H., Balaban M., Güler H. (2023). Kaggle. https://www.kaggle.com/datasets/suleyman32/pesplanus-two-class-dataset.

[4] Huang X., Shan J., Vaidya V. Lung nodule detection in CT using 3D convolutional neural networks. Proceedings of the 2017 IEEE 14th International Symposium on Biomedical Imaging (ISBI 2017).

[5] Gopatoti A., Vijayalakshmi P. (2022). CXGNet: A tri-phase chest X-ray image classification for COVID-19 diagnosis using deep CNN with enhanced grey-wolf optimizer. Biomed. Signal Process. Control..

[6] Litjens G., Kooi T., Bejnordi B.E., Setio A.A.A., Ciompi F., Ghafoorian M., van der Laak J.A.W.M., van Ginneken B., Sánchez C.I. (2017). A survey on deep learning in medical image analysis. Med. Image Anal..

[7] Talo M., Yildirim O., Baloglu U.B., Aydin G., Acharya U.R. (2019). Convolutional neural networks for multi-class brain disease detection using MRI images. Comput. Med Imaging Graph..

[8] Qin C., Yao D., Shi Y., Song Z. (2018). Computer-aided detection in chest radiography based on artificial intelligence: A survey. Biomed. Eng. Online.

[9] Ozturk T., Talo M., Yildirim E.A., Baloglu U.B., Yildirim O., Acharya U.R. (2020). Automated detection of COVID-19 cases using deep neural networks with X-ray images. Comput. Biol. Med..

[10] Tang A., Tam R., Cadrin-Chênevert A., Guest W., Chong J., Barfett J., Chepelev L., Cairns R., Mitchell J.R., Cicero M.D. (2018). Canadian Association of Radiologists white paper on artificial intelligence in radiology. Can. Assoc. Radiol. J..

[11] Khanna V.V., Chadaga K., Sampathila N., Prabhu S., Chadaga R., Umakanth S. (2022). Diagnosing COVID-19 using artificial intelligence: A comprehensive review. Netw. Model. Anal. Health Informatics Bioinform..

[12] Doğan K., Selçuk T., Yılmaz A. (2024). A Novel Model Based on CNN–ViT Fusion and Ensemble Learning for the Automatic Detection of Pes Planus. J. Clin. Med..

[13] Zhu J., Tan Y., Lin R., Miao J., Fan X., Zhu Y., Liang P., Gong J., He H. (2022). Efficient self-attention mechanism and structural distilling model for Alzheimer’s disease diagnosis. Comput. Biol. Med..

[14] Wolf T., Debut L., Sanh V., Chaumond J., Delangue C., Moi A., Cistac P., Rault T., Louf R., Funtowicz M. Transformers: State-of-the-art natural language processing. Proceedings of the 2020 Conference on Empirical Methods in Natural Language Processing: System Demonstrations.

[15] Guo M.-H., Liu Z.-N., Mu T.-J., Hu S.-M. (2022). Beyond self-attention: External attention using two linear layers for visual tasks. IEEE Trans. Pattern Anal. Mach. Intell..

[16] Qiu J., Mitra J., Ghose S., Dumas C., Yang J., Sarachan B., Judson M.A. (2024). A multichannel CT and radiomics-guided CNN-ViT (RadCT-CNNViT) ensemble network for diagnosis of pulmonary sarcoidosis. Diagnostics.

[17] Rahali A., Akhloufi M.A. (2023). End-to-end transformer-based models in textual-based NLP. AI.

[18] Bajaj D., Bharti U., Gupta I., Gupta P., Yadav A. (2024). GTMicro—Microservice identification approach based on deep NLP transformer model for greenfield developments. Int. J. Inf. Technol..

[19] Zhou Y. (2024). A serial semantic segmentation model based on encoder-decoder architecture. Knowledge-Based Syst..

[20] Al-Fahsi R.D.H., Prawirosoenoto A.N.F., Nugroho H.A., Ardiyanto I. (2024). GIVTED-Net: GhostNet-Mobile Involution ViT Encoder-Decoder Network for Lightweight Medical Image Segmentation. IEEE Access.

[21] Parvaiz A., Khalid M.A., Zafar R., Ameer H., Ali M., Fraz M.M. (2023). Vision Transformers in medical computer vision—A contemplative retrospection. Eng. Appl. Artif. Intell..

[22] Islam O., Kumer K., Akter S., Uddin M. Multi-Head Self-Attention Mechanisms in Vision Transformers for Retinal Image Classification. Proceedings of the 2024 IEEE International Conference on Computing, Applications and Systems (COMPAS).

[23] Dosovitskiy A., Beyer L., Kolesnikov A., Weissenborn D., Zhai X., Unterthiner T., Dehghani M., Minderer M., Heigold G., Gelly S. (2020). An image is worth 16x16 words: Transformers for image recognition at scale. arXiv.

[24] Zhou T., Niu Y., Lu H., Peng C., Guo Y., Zhou H. (2024). Vision transformer: To discover the “four secrets” of image patches. Inf. Fusion.

[25] Lee S.H., Lee S., Song B.C. (2021). Vision transformer for small-size datasets. arXiv.

[26] Katar O., Yildirim O. (2023). An explainable vision transformer model based white blood cells classification and localization. Diagnostics.

[27] Diker A., Sönmez Y., Özyurt F., Avcı E., Avcı D. (2021). Examination of the ECG signal classification technique DEA-ELM using deep convolutional neural network features. Multimedia Tools Appl..

[28] Diker A., Avci D., Avci E., Gedikpinar M. (2018). A new technique for ECG signal classification genetic algorithm Wavelet Kernel extreme learning machine. Optik.

[29] Reyad M., Sarhan A.M., Arafa M. (2023). A modified Adam algorithm for deep neural network optimization. Neural Comput. Appl..

[30] Lambora A., Gupta K., Chopra K. Genetic algorithm—A literature review. Proceedings of the 2019 international conference on Machine Learning, Big Data, Cloud and Parallel Computing (COMITCon).

[31] Fang J., Liu W., Chen L., Lauria S., Miron A., Liu X. (2023). A survey of algorithms, applications and trends for particle swarm optimization. Int. J. Netw. Dyn. Intell..

[32] Nogay H.S., Adeli H. (2023). Diagnostic of autism spectrum disorder based on structural brain MRI images using, grid search optimization, and convolutional neural networks. Biomed. Signal Process. Control..

